# Broad Target Metabolomics Revealed the Key Regulatory Mechanisms of the Effects of Trace Element Water-Soluble Fertilizer on the Growth of *Corylus heterophylla* × *Corylus avellana* Seedlings

**DOI:** 10.3390/genes16040373

**Published:** 2025-03-25

**Authors:** Weiqing Chen, Chao Ma, Mengjiong Zhao, Zaiguo Liu, De Zhang, Juan Lu, Jing Hao, Lei Wu

**Affiliations:** 1 Gansu Academy of Forestry, Lanzhou 730070, China; chaoma2024@126.com (C.M.); zmj.413@163.com (M.Z.); zaiguoliu@126.com (Z.L.); lujuan256@sina.com (J.L.); 18109447227@163.com (J.H.); wuballet@126.com (L.W.); 2 College of Horticulture, Gansu Agricultural University, Lanzhou 730070, China; 1073324010096@st.gsau.edu.cn

**Keywords:** *Corylus heterophylla* × *Corylus avellana*, broad target metabolomics, trace element water-soluble fertilizer, liquid chromatography–tandem mass spectrometry (LC-MS/MS) technology, flavonoids

## Abstract

Background: Scientific and rational fertilizer management can not only improve the yield and quality of hazelnut (*Corylus heterophylla* × *Corylus avellana*) but also reduce the negative impact on the environment. Methods: Liquid Chromatography–tandem Mass Spectrometry (LC-MS/MS) technology was used to reveal the contents of various metabolites in hazelnut seedlings, and differential metabolites were screened by principal component analysis (PCA) and partial least squares discriminant analysis (PLS-DA). Results: The results showed that a total of 178 up-regulated differential metabolites (Fold change > 1) and 175 down-regulated differential metabolites (Fold change < 1) were detected in 6 comparison groups (DWF0 vs. DWF4, DWF0 vs. DWF5, DWF0 vs. DWF6, DWF4 vs. DWF5, DWF4 vs. DWF6, DWF5 vs. DWF6). Interestingly, the flavonoid metabolic pathway was dramatically enriched, and it was involved in each fertilization combination. The metabolites of the flavonoid pathway in different fertilized and unfertilized groups were compared and analyzed, which displayed that metabolites tricetin, eriodictyol, garbanzol, apigenin, and biochanin A were significantly up-regulated, while garbanzol and astraglin were significantly down-regulated. More interestingly, the determination of flavonoid content and real-time fluorescence quantitative polymerase chain reaction (qRT-PCR) displayed that the application of trace element water-soluble fertilizer could significantly enhance the flavonoid content and the expression of genes related to the flavonoid biosynthesis pathway, such as phenylalanine ammonia-lyase (PAL) and cinnamate 4-hydroxylase (C4H), with the DWF4 treatment displaying the most significant values. Conclusions: Overall, the application of trace element water-soluble fertilizer (especially the DWF4 treatment) markedly affected the changes in key metabolites of the flavonoid pathway and the expression levels of key genes, thus promoting the growth and development of the hazelnut, which offers an important starting point for future analysis through genetic engineering.

## 1. Introduction

Hazelnut (*Corylus heterophylla* Fisch. × *C.avellana* L.), also known as mountain chestnut, is rich in nutrients such as protein, vitamins, and carbohydrates, and has always been known as the “king of nuts” [1,2]. At the same time, hazel trees possess well-developed underground roots that are densely intertwined, which contain high ecological benefits such as water retention and fertilizer retention [3]. The cultivation technology of hazelnuts in foreign countries has been highly refined, and the production level of hazelnuts in hazelnut-producing countries like Italy and the United States leads the world. These countries have implemented scientific fertilization, significantly enhancing the yield and quality of nuts [4]. High-quality hazelnut varieties were obtained through breeding experiments in Turkey. The fruits are large-sized, have high yields, and are suitable for roasting [5]. In Italy, hazelnut cultivation is mostly intensively managed, and the design of hazelnut orchards has been improved, with the dense planting method used for cultivation, which can greatly increase the yield and quality [6,7]. The selected hazelnut varieties have the unique advantage of ease of processing (round fruit shape, easy separation of the shell and seed kernel, and easy peeling of the seed kernel) [8]. Ninety percent of the hazelnuts produced and imported by Italy are used for processing, and its hazelnut-processed products include chocolate, pastries, candies, and ice cream, and processed products are not only sold at home, but also exported to other European countries [9]. The main chocolate variety of the famous Italian chocolate brand “Ferrero” is hazelnut chocolate [10]. Additionally, the development of the hazelnut industry in China began relatively late, but it has grown rapidly. From 2001 to 2019, the harvested area of hazelnuts increased from 7000 hm^2^ to 13,824 hm^2^; the yield of hazelnuts rose from 1.1 × 10^7^ kg to 2.9318 × 10^7^ kg. However, the growth rate in production is far from meeting the increase in consumer demand, and the import volume of hazelnuts has doubled in the past 10 years [11]. Therefore, the establishment of an efficient and scientific fertilization program is of great significance for the improvement of hazelnut yield and quality in China.

Basic fertilizers (nitrogen, phosphorus, and potassium) are the most important nutrients for trees, requiring sufficient fertilizer supply to achieve optimal growth, maximum yield, and high-quality nuts [12]. Several studies have shown that the mineral composition, protein, and ash content of hazelnuts are influenced by climate, variety, soil composition, fertilizer and irrigation use, cultivation methods, harvest year, and geographical origin [13,14,15,16]. Previous studies have suggested that the main metals, trace metals, and heavy metals in hazelnuts are K, P, Ca, Mg, Fe, Zn, Cu, Mn, and other elements [17,18]. Although the main mineral components of hazelnuts are known, there is little research on their cultivation techniques, especially fertilization. A good fertilization plan is necessary to achieve a high yield and high quality of hazelnuts. Research has shown that applying urea fertilizer twice underground at a rate of 50% enhances the quantitative and qualitative properties of hazelnuts. The results showed statistical increases in protein levels, grain proportions, nutrient concentrations in leaves, and hazelnut yield [19]. Meanwhile, Özenç et al. [20] found that applying different doses of iron fertilizer every year for three consecutive years could significantly affect the nut character and nut mineral composition, and increase total oil content, kernel percentage, and kernel weight. In summary, different doses of fertilizer display different effects on the quality improvement and nutrient accumulation of hazelnuts.

Therefore, this study used liquid chromatography and mass spectrometry combined with principal component analysis (PCA) and orthogonal partial least squares discriminant analysis (OPLS-DA) to elucidate the differences in metabolites among different fertilizer types and application amounts in hazelnuts, and to screen for identifying differential metabolites and determine the accumulation of differential metabolites in different fertilization processes. The optimal fertilization plan was selected, laying the foundation for the cultivation of high-quality seedlings in the later stage.

## 2. Plant Materials and Methods

The 2-year-old green stem cuttings of ’Davey’—a superior cultivar of European hybrid hazelnuts—were used as the experimental material. The experiment was conducted in the greenhouse at the Wuxingping Research Base of Gansu Provincial Forestry Academy from May 2023 onwards. Average temperature: 26 C, relative humidity: 50%. On 20 June 2023, 60 uniform and evenly growing seedlings were selected and transplanted into plastic pots (13 × 9 cm) filled with a mixture of soil (garden soil, perlite, and humus in a 2:1:1 volume ratio). Subsequently, they were then uniformly placed in the nursery for cultivation and management. Two weeks after transplanting, the experimental treatment was initiated. Fertilization treatment was carried out on 4 July 2023, and the experiment was conducted according to the same irrigation amount and the same irrigation cycle. The water control tray was placed at the bottom of the pot to achieve effective water and fertilizer control, and we made sure that the flowerpot was effectively water-resistant and maintained the same management and environmental conditions as far as possible. The fertilizers used in the test were A: water-soluble fertilizer of a large number of elements (N + P20s + K, 0 ≥ 55.0%, nutrient content 10-30-15, Zn + B = 0.4%~3.0%); the fertilizer is a powder, produced by Gansu Shikefeng Ecological Technology Co., LTD(Jinchang city, China); B: water-soluble fertilizer of trace elements (Amino acid ≥100 g/; Cu + Fe + Mn + Zn + B ≥ 20 g/L), developed and produced by Shaanxi Yinong Shangpin Agriculture Co., Ltd (Xi-An city, China); and C: humic acid fertilizer (Mineral source potassium fulvic acid, Stanley Agriculture Co., LTD (Linyi City, China)., mineral source fulvic acid (dry basis) >50%, potassium oxide K_2_O > 12%, moisture ≤ 15% (powder)), and the application form was water-fertilizer coupling. A total of 8 treatments were set up in the experiment (Table 1), 15 seedlings were selected as one replicate for each treatment, and a total of 3 biological replicates were performed. Each pot is watered 1 L each time. The fertilizer content of 1 L of water is shown in Table 1, which should be watered once every ten days. In addition, it should be noted that a large number of elemental water-soluble fertilizers and humic acid fertilizers have been found through previous studies to dissolve 1 g and 0.5 g in 1 L water, respectively, achieving the best hazelnut growth. This study was conducted to investigate the effects of water-soluble fertilizer on hazelnut growth and development.

## 3. Experimental Methods

### 3.1. Measurement of Photosynthetic Characteristics

As described by Lin et al. (2022), functional leaves of the same segment were selected and the indexes were determined by Li-6400 photosynthesometer (LI-COR, Lincoln, NE, USA), including net photosynthetic rate (Pn), stomatal conductance (Gs), transpiration rate (Tr), and intercellular CO_2_ concentration (Ci), which were repeated 3 times [21]. Subsequently, an Imaging-PAM chlorophyll fluorescence spectrometer along with Imaging WinGegE software (Walz, Effeltrich, ZQ-WALZ-010, Germany) was employed to determine initial fluorescence (F_0_), maximum photochemical efficiency (Fv/Fm), apparent photosynthetic electron transport rate (ETR), and actual quantum yield of PSII (ΦPSII).

The chlorophyll a (Chl a) and chlorophyll b (Chl b) of the leaves were measured according to the method described by Arnon et al. (1949) [22]. Absorbance was measured at wavelengths of 440 nm, 645 nm, and 663 nm. Chlorophyll a (Chl a) content was calculated as 12.21 A_663_–2.81 A_645_; chlorophyll b (Chl b) content as 20.13 A_645_–5.03 A_663._

### 3.2. Broad Target Metabolomics

The dry sample extraction method before extensive targeted metabolism was as follows: the 20 mg sample was taken, and 1000 uL 75% methanol aqueous solution (*V*/*V*) was added and ground at 35 Hz for 4 min and ultrasound for 5 min. This step was repeated twice, placed at 40 °C for 1 h, centrifuged at 12,000 rpm at 4 °C for 10–15 min, the supernatant was retained, and then filtered (0.22 μm) into the sample bottle.

Liquid chromatography–high-resolution mass spectrometry (LC-MS/MS) technology was used to perform metabolomic analysis. The chromatographic conditions were determined as follows: column: ACQUITY UPLC BEH C18 (100 mm × 2.1 mm, 1.7 μm); Column temperature: 45 °C; Mobile phase: A is water (containing 0.1% formic acid); B is methanol. The flow rate is 0.35 mL/min; the injection volume is set to 10 μL; Mass spectrometry conditions: ion source, ESI; positive and negative ion scanning modes are used for sample mass spectrometry signal acquisition.

### 3.3. Determination of Flavonoid Content

The flavonoid content was determined using the NaNO_2_-Al(NO_3_)_3_-NaOH colorimetric method. Dry hazelnut leaves were weighed to add 60% (volume fraction) ethanol, ultrasound for 30 min (40 kHz), centrifuged at 10,000 r/min for 15 min, the supernatant was combined, and then paused for testing. Then, 0.5 mL of the extract was taken into a 10 mL volumetric bottle, and 0.5 mL 5% NaNO_2_ solution, 0.5 mL 10% Al(NO_3_)_3_ solution, 4.0 mL (1 mol/L) NaOH solution, and 60% ethanol were added to the volume, respectively. The absorbance of the sample solution at 510 nm wavelength was determined and repeated 3 times.

### 3.4. Quantitative Real-Time PCR

Total RNA was extracted from the samples using an RNA extraction kit (BioTeke Corporation, Beijing, China). TaKaRa’s PrimeScriptTM RT reagent Kit with gDNA Eraser (Perfect Real Time) was performed for reverse transcription. Primers were designed with Shanghai Sangon Biological Engineering Co., LTD (Shanghai city, China) (Table 2). Meanwhile, cDNA of *Corylus heterophylla* × *C. avellana* plantlets was regarded as the template and GAPDH as a reference for quantitative real-time PCR. Quantitative data analysis was performed using the 2^−ΔΔCt^ method. The reaction system was composed of TB Green™ Premix Ex Taq II, 10 μmol L^−1^ upstream primers, 10 μmol L^−1^ downstream primers, cDNA template, and dd H_2_O at doses of 10, 1, 1, 2, and 6 μL, respectively. Reaction conditions were as follows: pre-denaturation at 95 °C for 3 min, denaturation at 95 °C for 5 s, annealing at 58 °C for 30 s, extension at 72 °C for 30 s, repeated for 40 cycles. Each sample was repeated three times.

### 3.5. Data Preprocessing and Statistical Analysis

Qualitative analysis of the raw data was used for the Human Metabolome Database (HMDB) database (V4.0) [23]. The software SIMCA (V14.1) was used to perform multivariate statistical analysis on preprocessed data [24,25]. Orthogonal partial least squares discriminant analysis (OPLS-DA) was used to remove irrelevant differences and screen for differential variables to screen for metabolic differences [26]. Finally, based on the KEGG database (https://www.kegg.jp/kegg/compound (accessed on 11 October 2024)) metabolic pathway enrichment analysis was performed on differential metabolites. The clustering analysis of differential metabolites was visualized using TBtools software (version number: v2.056). Additionally, experimental data are presented as the mean ± sd of three independent replicates. Data were analyzed via one-way ANOVA (*p* < 0.05). SPSS (version 22.0, IBM, Armonk, NY, USA) was used for statistical analysis, and Origin 7.0 software was used for data processing (OriginLab, Hampton, MA, USA).

## 4. Results

### 4.1. Effects of Different Doses of Trace Element Water-Soluble Fertilizers on Photosynthetic Parameters of Hazelnut Leaves

**Figure 1:**
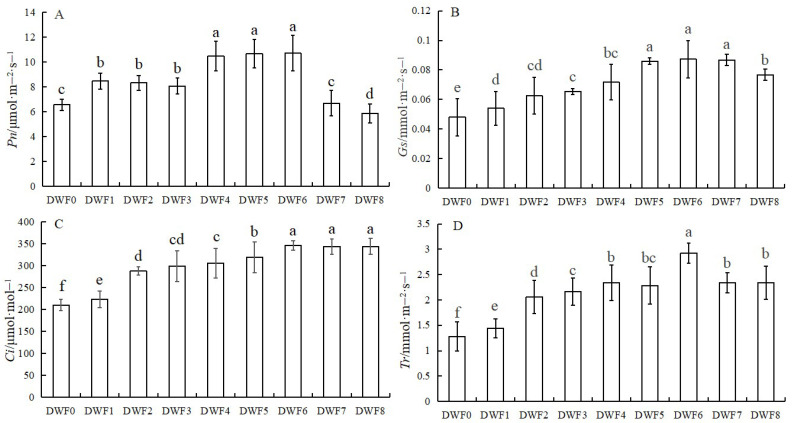
Effects of different doses of trace element water-soluble fertilizers on photosynthetic parameters of hazelnut leaves: (**A**) Net photosynthesis (*P*n), (**B**) Stomatal conductance (*G*s), (**C**) Intercellular CO_2_ concentration (*C*i), (**D**) Transpiration rate (*T*r). Different lowercase letters stand for the significant difference at the 0.05 level.

As illustrated in Figure 1, the photosynthetic parameters of the treated groups exhibited a significant increase compared to the control group without the application of trace element water-soluble fertilizer. Notably, treatment group DWF6 demonstrated the most substantial improvements in photosynthetic parameters. Specifically, the net photosynthetic rate (*P*n) of DWF6 increased by 63.01% compared to DWF0 (Figure 1A). The stomatal conductance (*G*s) and the intercellular CO_2_ showed increases of 81.88% and 64.19%, respectively (Figure 1B,C). In addition, the transpiration rate (*T*r) also reaches its maximum in the DWF6 treatment, followed by the DWF4 treatment (Figure 1D).

### 4.2. Effects of Different Doses of Trace Element Water-Soluble Fertilizers on the Fluorescence Parameters of Hazelnut Leaves

**Figure 2:**
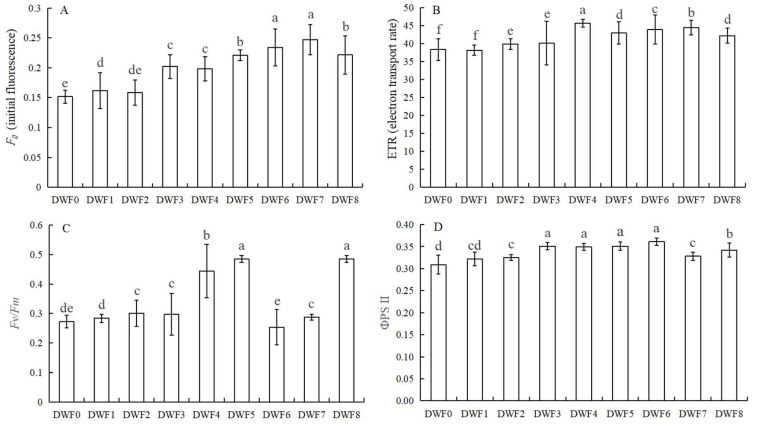
Effects of different doses of trace element water-soluble fertilizers on the fluorescence parameters of hazelnut leaves: (**A**) The initial fluorescence (*F*_0_), (**B**) Electron transport rate (ETR), (**C**) Maximal photochemical efficiency (*F*_v_/*F*_m_), (**D**) ΦPSII: actual photochemical efficiency. Different lowercase letters stand for the significant difference at the 0.05 level.

As shown in Figure 2, the fluorescence parameters of the treatment group were significantly improved compared with the control group without applying water-soluble fertilizer with trace elements. Among them, F0 reaches the maximum value in DWF7, which is 0.25, followed by DWF6, which is 0.23 (Figure 2A). The ETR reaches the maximum value of 45.67 in the DWF4 treatment, which is 19.18% higher than that in DWF0 (Figure 2B). Maximal photochemical efficiency (*F*_v_/*F*_m_) reached the maximum value at DWF5, which was 0.485, an increase of 78.30% compared with DWF0 (Figure 2C). Actual photochemical efficiency (ΦPSII) reached the maximum value of 0.36 in the DWF6 treatment, which was 16.12% higher than that in the DWF0 treatment (Figure 2D).

### 4.3. Effects of Different Doses of Trace Element Water-Soluble Fertilizers on Photosynthetic Pigments in Hazelnut Leaves

**Figure 3:**
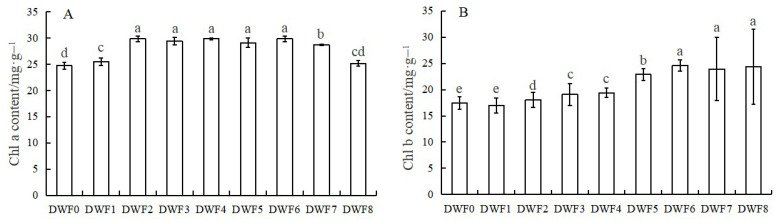
Effects of different doses of trace element water-soluble fertilizers on photosynthetic pigments in hazelnut leaves: (**A**) Chlorophyll a content (Chl a), (**B**) Chlorophyll b content (Chl b). Different lowercase letters stand for the significant difference at the 0.05 level.

As shown in Figure 3, the Chlorophyll a content reached the maximum value in the DWF4 treatment, which was 29.85 mg·g^−1^, followed by DWF6 and DWF2. The Chlorophyll b content had the highest value in DWF6, which was 24.60 mg·g^−1^, followed by DWF8 and DWF7.

### 4.4. Metabolic Trajectory of Hazelnut Leaves Treated with Different Doses of Trace Element Water-Soluble Fertilizers

**Figure 4:**
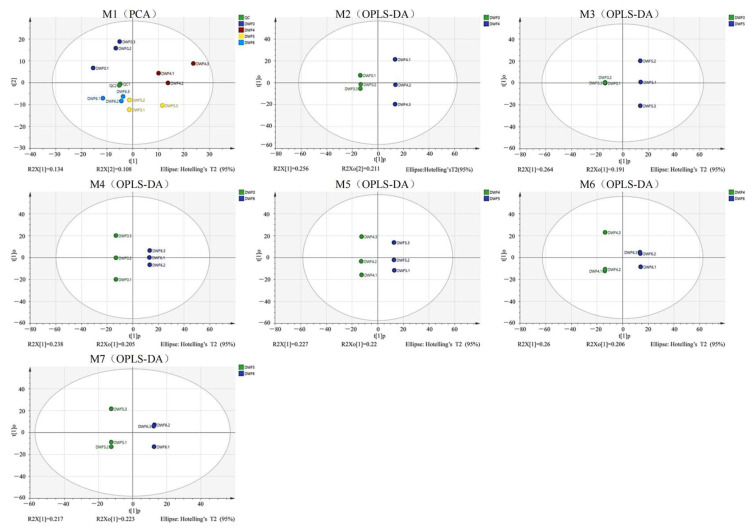
Metabolomic trajectory of hazelnut leaves under different fertilization treatments using the M1 principal component analysis (PCA) scoring chart. M2, M3, M4, M5, M6, and M5 are six processed orthogonal partial least squares discriminant analyses (OPLS-DA).

Based on the above physiological data, we found that when the amount of trace water-soluble fertilizer reached a certain level (DWF6 treatment), and then continued to increase the dose of this type of fertilizer, the photosynthetic parameters, fluorescence parameters, and chlorophyll content of hazelnuts leaves had no significant changes. Therefore, DWF0, DWF4, DWF5, and DWF6 were selected for metabolomics analysis. QC (Quality Control) samples are prepared by mixing all sample extracts and can be used to analyze the repeatability of samples under the same processing conditions. After performing adaptive (UV) conversion on the data, the PCA plots of all samples and QC (Figure 4 M1) show that the QC samples in the plot are clustered and possess good repeatability, indicating the stability of the system. In order to distinguish the six treatments to the maximum extent, we used SIMCA software for orthogonal partial least squares discriminant analysis (OPLS-DA) modeling analysis. OPLS-DA combines orthogonal signal correction (OSC) and partial least squares discriminant analysis (PLS-DA) methods, resulting in better clustering between parallel samples on OPLS-DA (Figure 1 M2, M3, M4, M5, M6, M7). And all samples are within the 95% confidence interval (Hotelling’s T-Squared ellipse), indicating a significant difference between the two groups of samples, which can be used for fitness and prediction.

### 4.5. Metabolite Analysis of Hazelnut Leaves

**Figure 5:**
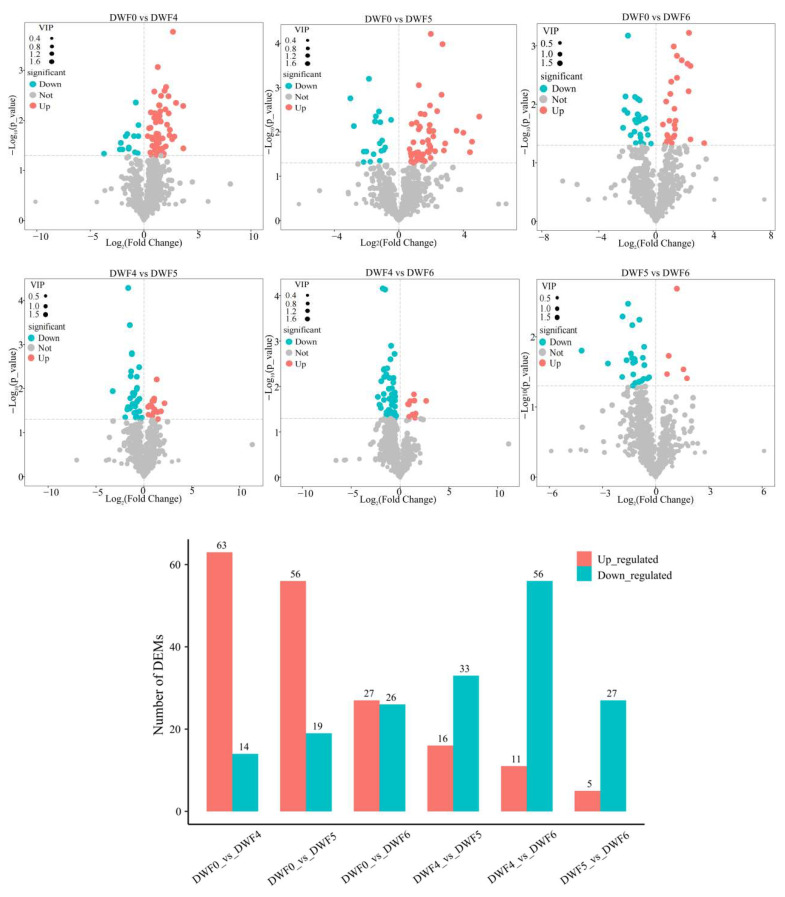
Volcanic diagram and column chart of differential metabolites in hazelnut leaves from 6 comparison groups. The red dots represent the up-regulation of metabolites, the green dots represent the down-regulation of metabolites, and the gray dots represent no difference.

Based on the importance projection (VIP) variable analysis results of the OPLS-DA model for multivariate analysis, combined with the *p*-value and fold change of the Student’s *t*-test for univariate analysis, differential metabolites were screened. Using *p*-value < 0.05 and VIP > 1 as criteria to compare the differential metabolites between two groups, in order to facilitate metabolite screening, volcano plots were used to visualize the *p*-value and difference fold analysis, as shown in Figure 5. Statistical analysis of differential metabolites in volcano maps of six comparison groups revealed that 63, 56, and 27 up-regulated metabolites and 14, 19, and 26 down-regulated metabolites were detected, respectively, in the DWF0 compared to the DWF4, DWF5, and DWF6 groups. When DWF4 was used as a control, the comparison groups DWF5 and DWF6 detected 16 and 11 up-regulated metabolites, and 33 and 56 down-regulated metabolites; when DWF5 was used as a control, there were 5 up-regulated and 27 down-regulated metabolites in DWF6, respectively. In order to further analyze and clarify the main metabolites generated during the fertilization process, we presented the top 15 metabolites (*p*-value < 0.05) of each treatment group (Table 3). The differentially expressed metabolites mainly include flavonoids, alkaloids, nucleotides, coumarins, terpenes, and other metabolites.

### 4.6. Hierarchical Clustering Analysis of Metabolic Components in Leaves of Hazelnut at Different Doses of Trace Element Water-Soluble Fertilizers

**Figure 6:**
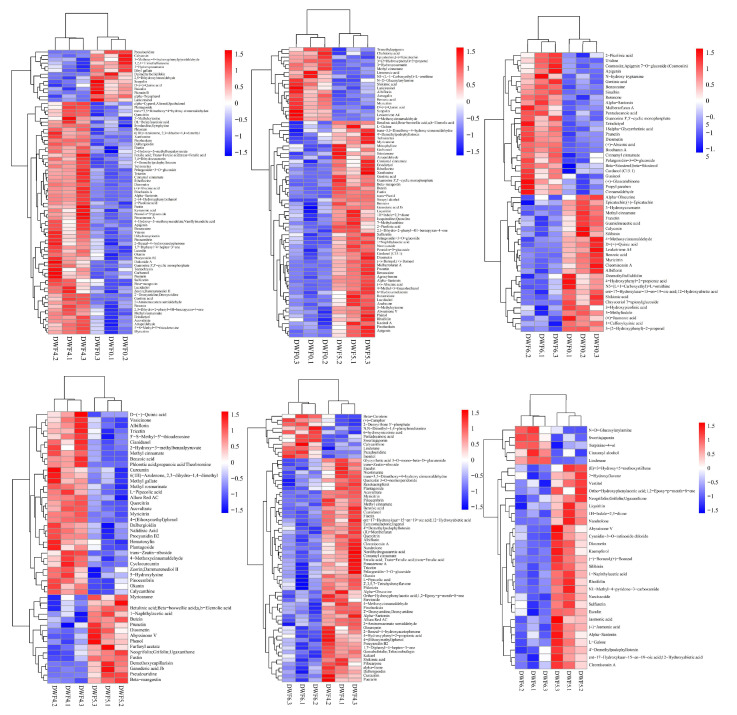
Hierarchical clustering analysis of metabolic components in leaves of hazelnut treated with different doses of micro-water-soluble fertilizers.

To study the effects of water-soluble fertilizers with varying doses of trace elements on metabolites in hazelnut leaves, pairwise analysis and visualization of metabolites after one fertilization treatment and three fertilization treatments were conducted through hierarchical cluster analysis (Figure 6). The findings indicated that numerous key metabolites were significantly up-regulated following fertilization compared to the no-fertilization treatment. Specifically, metabolites of the flavonoid biosynthesis pathway, such as procyanidin and tricetin, were significantly enriched and up-regulated in DWF4 vs. DWF0. Similarly, metabolites in DWF5 vs. DWF0 and DWF6 vs. DWF0 within the flavonoid biosynthesis pathway were also significantly enriched. Additionally, alkaloids, nucleotides, coumarins, terpenes, and other metabolites were up-regulated. More interestingly, there were also similarities and differences in metabolites between each fertilization treatment (DWF4 vs. DWF5, DWF5 vs. DWF6, DWF4 vs. DWF6). On the one hand, flavonoid metabolites were significantly accumulated after fertilization, and the regulation of metabolites by different doses of water-soluble fertilizers may be different.

### 4.7. Pathway Mapping and Metabolite-to-Metabolite Network Visualization

**Figure 7:**
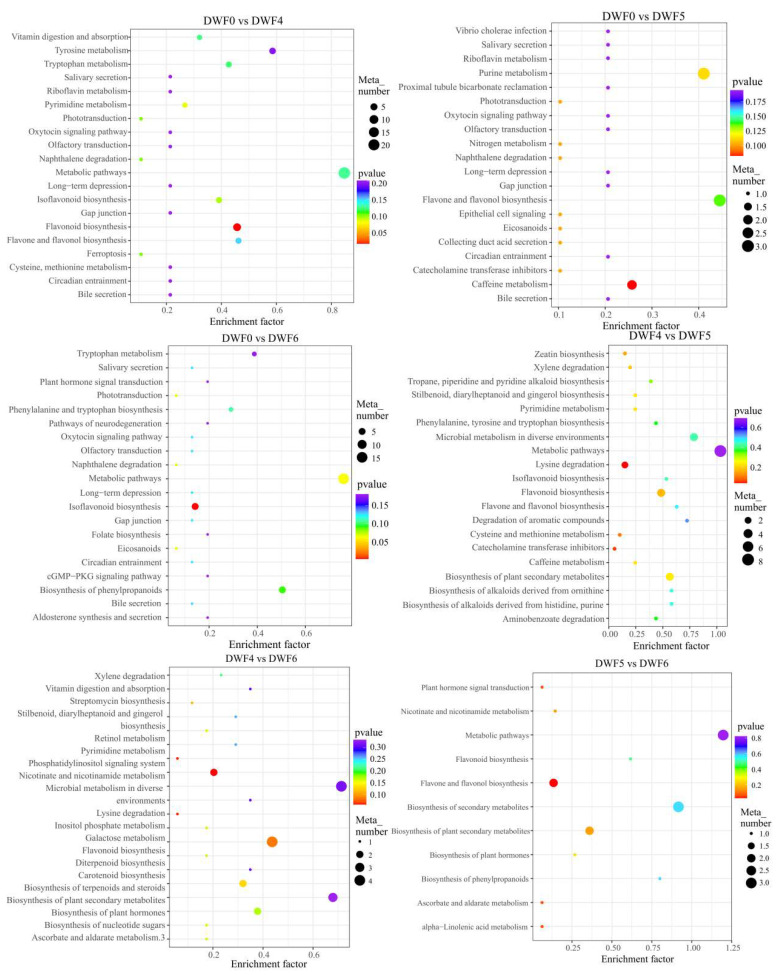
Bubble plot of differential metabolite enrichment in hazelnut leaves. The horizontal axis represents the Rich Factor corresponding to each pathway, with a larger value indicating a greater degree of enrichment. The vertical axis represents the pathway name, and the color of the dots reflects the *p*-value size. The redder the value, the more significant the enrichment. The size of the dots represents the number of enriched differential metabolites.

The KEGG database is a powerful tool for identifying the biological processes involved. Based on logP values and pathway impact scores, each pathway enriched DEM under different fertilization treatments (Figure 7, Table 4). It is worth noting that under the DWF0 vs. DWF4 treatment, there were significant differences in flavonoid biosynthesis, pyrimidine metabolism, and isoflavonoid biosynthesis compared to other metabolic pathways in the leaves. Then, important metabolites were enriched in DWF0 vs. DWF5, including caffeine metabolism, catecholamine transferase inhibitors, collecting duct acid secretion, epithelial cell signaling in helicobacter pylori infection, eicosanoids, naphthaline degradation, phototransduction, purine metabolism, and flavone and flavonol synthesis. However, in DWF0 vs. DWF6, isoflavonoid biosynthesis is significantly more abundant than other metabolic pathways. In DWF4 vs. DWF5, it was mainly enriched in lysine degradation and catecholamine transferase inhibitors (*p* < 0.05). Under the DWF4 vs. DWF6 treatment, the main metabolic pathways were enriched in nicotine and nicotinamide metabolism, inositol phosphate metabolism, phosphatidylinositol signaling system, and flavonoid biosynthesis. Under the DWF5 vs. DWF6 treatment, the differential metabolites of flavone and flavonol biosynthesis were more significant than other metabolic pathways. Overall, different fertilization stages have significantly enhanced metabolic pathways and related metabolites, and flavonoid metabolism is involved in each fertilization stage, and each stage shows relatively significant effects. This indicates that the elucidation of these metabolic pathways will complete our understanding of hazelnut leaf metabolites.

To further explore the molecular regulatory mechanisms of flavonoid metabolism pathways (flavonoid biosynthesis, isoflavonoid biosynthesis, and flavone and flavonol biosynthesis) on the effects of different fertilization treatments on hazelnut leaves. We analyzed the specific metabolites in the enriched metabolic pathways (Table 4) and found that the flavonoid biosynthesis pathway was enriched in each treatment, with 11 differential metabolites enriched. Up-regulated metabolites included tricetin, pinocembrin, eriodictyol, garbanzol, phlorizin, luteolin, and apigenin. In the isoflavonoid biosynthesis pathway, except for DWF4 vs. DWF6 and DWF5 vs. DWF6, which were not enriched, they were enriched in all other treatments, and there were five differentially expressed metabolites enriched, namely, calycosin, biochanin A, apigenin, prunetin, and rotenone. Among them, the up-regulated metabolites were biochanin A and apigenin. There are six metabolites involved in the flavonoid biosynthesis pathway, namely, quercillin, luteolin, apigenin, astrolin, rhoifolin, and kaempferol. The up-regulated metabolites are quercillin, luteolin, and apigenin. The results indicate that the response of plants to different fertilization treatments may be caused by the accumulation of metabolites.

### 4.8. Cluster Analysis of Differential Flavonoid Metabolites

**Figure 8:**
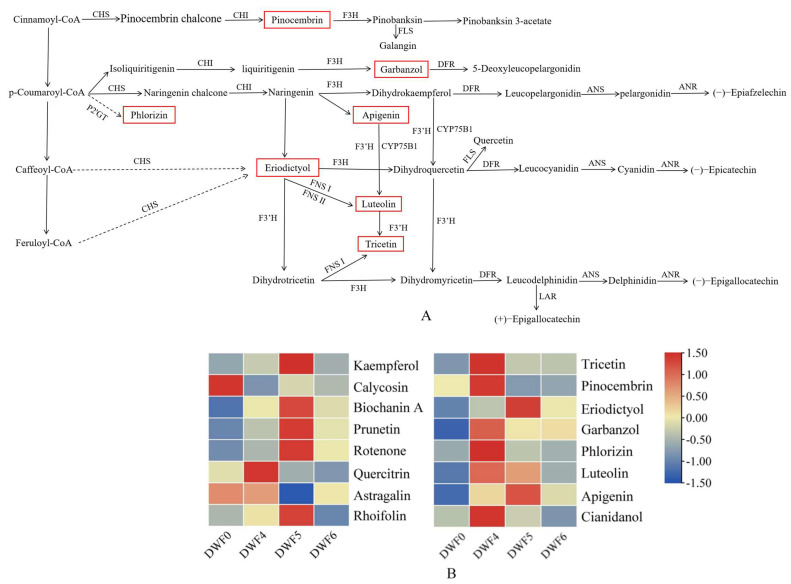
Cluster analysis of differential flavonoid metabolites mapped onto the KEGG pathway: (**A**) flavonoid pathway (**B**) key metabolites in the flavonoid pathway. Note: Metabolites in the red box in Figure 8A are represented as up-regulated metabolites.

In order to analyze the changes of different metabolites in flavonoids in hazelnut leaves under different fertilization treatments, cluster analysis was conducted on the differential flavonoid metabolites. The results showed that there were significant differences in flavonoid metabolites among the samples DWF0, DWF4, DWF5, and DWF6 (Figure 8). Mapping differential metabolites to the KEGG synthesis pathway, only 16 differential metabolites were annotated in the flavonoid biosynthesis pathway (flavonoid biosynthesis, isoflavonoid biosynthesis, and flavone and flavonol biosynthesis) (Table 5). Nine flavonoid metabolites were annotated on the synthesis pathway of flavonoid biosynthesis. Five flavonoid metabolites were annotated on the isoflavonoid biosynthesis pathway. There are six flavonoid metabolites involved in the synthesis pathway of flavonoid and flavonol biosynthesis. Cluster analysis was conducted on 16 differentially expressed flavonoids, and the results showed that compared to the DWF0 treatment, the accumulation of each flavonoid metabolite was higher in the DWF4 and DWF5 treatments. There were eight metabolites significantly accumulated in each of the DWF4 and DWF5 treatments, and in the DWF4 treatment, they were tricetin, pinocembrin, garbanzol, phlorizin, luteolin, cianidanol, quercitrin, and astrolin, respectively. In the DWF5 treatment, there were eriodictyol, luteolin, apigenin, kaempferol, biochanin A, prunetin, rotenone, and rhoifolin, respectively. However, under the DWF6 fertilization treatment, the accumulation of metabolites was significantly lower than that of DWF4 and DWF5. This indicates that the application of DWF4 and DWF5 fertilizers is more conducive to the accumulation of metabolites.

### 4.9. Effects of Different Fertilization Treatments on Flavonoid Content and Expression Levels of Key Genes in Hazelnut Leaves

**Figure 9:**
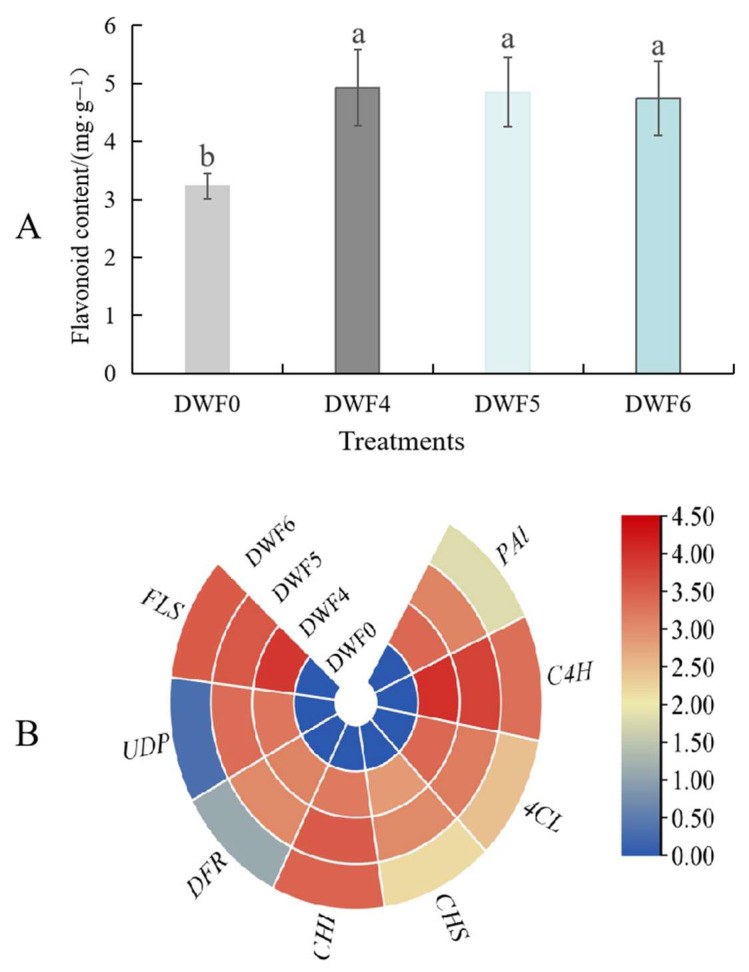
Flavonoid content and expression levels of key genes in hazelnut leaves and under different fertilization treatments: (**A**) Flavonoid contents (**B**) key genes in the flavonoid pathway. PAL: phenylalanine ammonia-lyase; C4H: cinnamate 4-hydroxylase; 4CL: p-coumaroyl coenzyme A ligase; CHS: chalcone synthase; CHI: chalcone isomerase; DFR: dihydroflavonol 4-reductase; UDP: uridine diphosphate; FLS: flavonol synthase. Different lowercase letters stand for the significant difference at the 0.05 level.

As shown in Figure 9, the flavonoid content of hazelnut leaves significantly increased under different fertilization treatments compared to the control (*p* < 0.05), and the DWF4 and DWF5 fertilization treatments performed better, while DWF6 performed worse. The above results indicate that different fertilization treatments have significant differences in the changes in leaf flavonoid content. However, the accumulation of flavonoid content was the best under DWF4 and DWF5 fertilization treatments, which increased by 53.12% and 51.86%, respectively, compared to DWF0.

The expression levels of key genes of the flavonoid biosynthesis pathway under various fertilization treatments (*p* < 0.05) are presented in Figure 9B. The findings reveal that the expression levels of key genes such as PAL, C4H, 4CL, CHS, CHI, DFR, UDP, and FLS were elevated compared to those of the non-fertilized treatment. Among them, the expression changes of the C4H, CHI, and FLS genes were the most remarkable. These results imply that the trace element water-soluble fertilizer can enhance the accumulation of flavonoids by promoting the expression of key genes in the flavonoid biosynthesis pathway.

## 5. Discussions

Hazelnut (*Corylus heterophylla* × *C. avellana*), as an important woody edible oil tree species with abundant resources and tolerance to drought and barrenness, has developed rapidly in China since 1980 [27]. However, excessive fertilization is common in most hazelnut orchards, and long-term excessive fertilization will lead to the deterioration of soil physical and chemical properties, and significant changes in cultivated land microbial community, and will thus hinder the growth of hazelnut and limit the yield growth potential. Therefore, a reasonable application amount and fertilization method can increase the yield and quality of hazelnuts and improve the utilization rate of water and fertilizer.

Chlorophyll, the primary pigment responsible for photosynthesis in plants, is a family of lipid-soluble pigments located within the thylakoid membrane, which plays a crucial role in the absorption, transfer, and transformation of light energy [28]. This study demonstrated that the application of trace element water-soluble fertilizer significantly increased the concentrations of chlorophyll a and chlorophyll b in hazelnut leaves compared to no fertilizer treatment, particularly with the DWF6 treatment, which is consistent with the findings of Mahesh and Raja (2015) [29]. Their findings suggested that water-soluble fertilizer can maintain a dynamic equilibrium between chlorophyll synthesis and degradation. It is believed that the chlorophyll content can reflect the strength of plant photosynthesis, and the strength of photosynthesis can be used as an important index to judge the strength of plant growth and stress resistance [30]. The results showed that the four photosynthetic parameters, P_n_, G_s_, C_i_, and T_r_ increased significantly after the application of trace water-soluble fertilizer, indicating that hazelnuts could better utilize chlorophyll to absorb light energy, convert carbon dioxide and water into organic matter, and provide nutrients for the growth of hazelnuts themselves, which is consistent with the finding of Guo (2021) [31]. He confirmed that the net photosynthetic rate, stomatal conductance, and transpiration rate of the leaves of the rabbiteye blueberry (*Vaccinium virgatum*) were significantly increased, and the photosynthetic capacity was finally improved under a certain amount of water and fertilizer supply. In addition, chlorophyll fluorescence parameters can directly reflect the internal effects of stress on photosynthesis [32]. The results showed that the *F*_0_, *F*_v_/*F*_m_, ETR, and ΦPSII of hazel leaves were gradually increased, indicating that the micro-water-soluble fertilizer could strengthen the photosynthetic system, enhance the utilization rate of light energy, and enhance the photosynthetic electron transfer activity. Interestingly, we found that when the amount of trace water-soluble fertilizer reached a certain level (DWF6 treatment), and then the amount of this type of fertilizer continued to increase, the photosynthetic parameters, fluorescence parameters, and chlorophyll content of hazelnut leaves did not change significantly. Therefore, DWF0, DWF4, DWF5, and DWF6 were selected for metabolomics analysis.

Metabolomics, a discipline that emerged after genomics and proteomics, constitutes an essential part of systems biology. Genomics and proteomics respectively explore the activities of life at the gene and protein level [33]. Nevertheless, numerous life activities within cells are associated with metabolites, such as cell signaling and energy transmission, which are regulated by metabolites. In this study, Liquid Chromatography–tandem Mass Spectrometry (LC-MS/MS) technology was used in this experiment to analyze the differences in small molecule metabolites and KEGG pathways in hazelnut leaves, and to explore the effects of trace element water-soluble fertilizer on hazelnut growth and development at the molecular level, providing a theoretical basis for high-yield cultivation in hazelnut orchards. The biosynthesis pathway of flavonoids was significantly enriched, and key metabolites such as tricetin, pinocembrin, biochanin A, apigenin, quercitrin, and luteolin were precisely identified based on the KEGG screening results.

Flavonoids are a class of secondary metabolites with a basic skeleton of C6-C3-C6. They are a type of phenolic compound composed of two benzene rings connected by three carbon atoms, and their biosynthesis is mainly achieved through the phenylpropane metabolic pathway [34]. Flavonoids have the functions of enhancing photosynthesis, promoting the growth and development of roots, stems, and leaves, and have many biological activities such as anti-insect, anti-oxidation, and regulating endocrine and metabolism in vivo [35,36]. Therefore, accurate quantification of flavonoids in samples such as fruits, vegetables, flowers, medicinal plants, and food can help understand plant growth and development and stress resistance mechanisms, and provide a theoretical basis for the impact of different food processing methods on nutritional value. This study compared six different fertilization treatments (DWF0 vs. DWF4, DWF0 vs. DWF5, DWF0 vs. DWF6, DWF4 vs. DWF5, DWF4 vs. DWF6, and DWF5 vs. DWF6) and analyzed the differences in the types and contents of metabolites in hazelnut leaves. As a result, it was found that 353 up- and down-regulated metabolites were screened among the hazelnut comparison groups under fertilization treatment, and significant changes were observed in the content of flavonoids, which may be due to the regulation of key enzyme activities involved in flavonoid synthesis. Previous studies found that flavonol metabolites such as quercetin and dihydromyricetin were significantly elevated in *Brassica napus* seedlings after cold stress [37]. In apples (*Malus domestica*), the accumulation of flavonols promotes the removal of reactive oxygen species (ROS) and the survival of plants under dry conditions [38]. In tomato (*Solanum lycopersicum*), flavonols can reduce ROS accumulation, regulate ABA-dependent ROS burst in guard cells, promote stomatal opening, and regulate leaf gas exchange [39]. In short, existing research has shown that flavonoids enhance the tolerance of certain plants to growth and development. In the present study, it was found that metabolites such as tricetin, pinocembrin, biochanin A, apigenin, quercitin, and luteolin were involved in biosynthesis. This is similar to the results obtained by Lelli et al. in identifying flavonoid metabolites in two hazelnut varieties (Tonda Gentile Romana and Tonda di Giffoni) [40]. Among them, tricetin, as a dietary flavonoid, is currently mainly focused on research into various cancers. It has shown anti-metastatic activity in osteosarcoma and glioblastoma cells [41,42,43]. In this study, tricetin significantly accumulated under different fertilization treatments, suggesting that it may play a crucial role in the early development of hazelnuts. Quercitrin is found in onions (*Allium cepa*) [44], broccoli (*Brassica oleracea*) [45], apples (*Malus domestica*) [46], tea (*Camellia sinensis*) [47], and red wine [48]. It is a water-soluble plant pigment with high antioxidant and anti-inflammatory activity [49]. Isoquercitrin can improve energy metabolism and enhance plant antioxidant capacity [50]. Although these substances are present in the metabolic pathway of flavonoids in hazelnuts, there is currently no specific research indicating whether these substances can be more effectively applied to people’s food and nutritional health. This provides a new direction and approach for our future research. In addition, we also identified flavonoids such as apigenin and luteolin, which showed the most drastic changes in hazelnuts under different fertilization treatments. Many variants of apigenin and luteolin have been found in hazelnuts, and these variants undergo changes in growth and development stages during different fertilization processes, such as apigenin 7-O-glucoside and luteolin-6-C-glucoside. This indicates that flavonoid compounds play a very important role in hazelnuts and have further research value. More interestingly, the application of water-soluble fertilizer with trace elements observably enhanced the flavonoid content and the expression level of PAL, C4H, 4CL, CHS, CHI, DFR, UDP, and FLS genes related to the flavonoid biosynthesis pathway, among which the DWF4 treatment had the most significant effect. In summary, hazelnuts, as an important nut resource, contain various flavonoids in the body of the nut. Alterations of these compounds subsequent to the application of the fertilizer might be associated with the growth, development, physiological metabolism, and environmental adaptation of hazelnuts.

## 6. Conclusions

Liquid chromatography–tandem mass spectrometry technology was used to identify metabolites of hazelnut leaves treated with different doses of water-soluble fertilizer. A total of 178 up-regulated and 175 down-regulated metabolites were detected. KEGG enrichment analysis showed that the pathway of flavonoid metabolism was significantly enriched after the application of trace water-soluble fertilizer, and the metabolites tricetin, eriodictyol, garbanzol, apigenin, and biochanin A in the pathway were significantly up-regulated. In addition, garbanzol and astraglin were significantly reduced. Meanwhile, the application of water-soluble fertilizer with trace elements observably enhanced the flavonoid content and the expression level of PAL, C4H, 4CL, CHS, CHI, DFR, UDP, and FLS genes related to flavonoid biosynthesis pathway, among which the DWF4 treatment had the most significant effect. In summary, the application of water-soluble fertilizer with trace elements (especially the DWF4 treatment) dramatically affected the changes in key metabolites of the flavonoid pathway tricetin, eriodictyol, apigenin, and the expression levels of key genes, thus promoting the growth and development of hazelnuts, which provided a theoretical basis for the efficient utilization of hazelnuts’ fertilizer.

## Figures and Tables

**Table 1:** Three kinds of (A, B, C) fertilizer combinations: A: water-soluble fertilizer of a large number of elements; B: water-soluble fertilizer of trace elements; C: humic acid fertilizer.

Group	Fertilization Treatment (g/L): A + B + C	Group	Fertilization Treatment (g/L): A + B + C
DWF0	0 +0 + 0	DWF5	1 + 1 + 0.5
DWF1	1 + 0.1 + 0.5	DWF6	1 + 1.5 + 0.5
DWF2	1 + 0.2 + 0.5	DWF7	1 + 2 + 0.5
DWF3	1 + 0.4 + 0.5	DWF8	1 + 2.5 + 0.5
DWF4	1 + 0.5 + 0.5		

**Table 2:** List of primers for real-time quantitative PCR analysis.

Gene Name	Primer Sequence (5′-3′)	
	Forward Primer	Reverse Primer
P-coumaroyl coenzyme A ligase (4CL)	TTAGCAAGCTCGGAATTCGGAAGG	CTCGGCGGCGGTGAAGAAAG
Flavonol synthase (FLS)	GCGTGAGGTGGCGGATAAACTG	GCGTTAGATCAGGCTGAGGACATG
Uridine diphosphate (UDP)	TGTTTCCGAATCTCCTGCCCATTG	ACCGAGCGAGTTGCTTGTTGATC
Dihydroflavonol 4-reductase (DFR)	GCTCTGCGTGCGATGCTACC	GCCTCCAAGTTCTCGTCCACATC
Chalcone isomerase (CHI)	GGCTCCACCAATACCTTGTTCCTC	ACGGCGGTATCCTCCAAGTACAC
Chalcone synthase (CHS)	AGACATAGTGGTGGCGGAGGTG	CGAGGTGGGTGATCTTGGATTTGG
Cinnamate 4-hydroxylase (C4H)	GTGTACGGTGAGCATTGGAGGAAG	CGCCTCTGGGTTCTTCTTCACATC
Phenylalanine ammonia-lyase (PAL)	CATATCCCAGGTGGCAGCGATTG	CGCCCTGCTTGGTGCTCTTG
Glyceraldehyde-3-phosphate dehydrogenase (GAPDH)	TGAGGGCAAGGTGAAGGGTATCTT	TCAAGTCAACCACACGCGTACTGT

**Table 3:** Relative concentration and fold changes of the top 15 major metabolites (*p* < 0.05) in hazelnuts before each treatment.

Treatments	Metabolites	VIP	*p*-Value	Fold Change
DWF0 vs. DWF4	Diosmetin	1.72376	1.69 × 10^−4^	6.56 × 10^0^
	Pinobanksin	1.72496	8.58 × 10^−4^	2.46 × 10^0^
	Telmisartan	1.68743	2.15 × 10^−3^	4.21 × 10^0^
	Dalbergioidin	1.69662	2.54 × 10^−3^	3.82 × 10^0^
	2-Aminomuconate semialdehyde	1.68287	2.65 × 10^−3^	1.96 × 10^0^
	Cinnamyl cinnamate	1.67804	3.22 × 10^−3^	3.04 × 10^0^
	Alpha-Santonin	1.7137	3.29 × 10^−3^	4.91 × 10^0^
	alpha-Tocopherol	1.67998	4.38 × 10^−3^	5.92 × 10^−1^
	2-(4-Hydroxyphenyl)ethanol	1.71552	4.46 × 10^−3^	8.00 × 10^0^
	Riboflavin	1.6886	4.98 × 10^−3^	2.66 × 10^0^
	Phlorizin	1.66599	4.98 × 10^−3^	2.74 × 10^0^
	Xanthosine	1.70745	5.14 × 10^−3^	1.27 × 10^1^
	Pelargonidin-3-O-glucoside	1.64755	6.03 × 10^−3^	4.03 × 10^0^
	trans-3,5-Dimethoxy-4-hydroxy cinnamaldehyde	1.62291	6.59 × 10^−3^	2.89 × 10^0^
	3,4-Dihydrocoumarin	1.64793	6.91 × 10^−3^	1.57 × 10^0^
DWF0 vs. DWF5	Butein	1.71868	6.14 × 10^−5^	3.96 × 10^0^
	Fustin	1.72626	1.04 × 10^−4^	6.55 × 10^0^
	3-(2-Hydroxyphenyl)-2-propenal	1.70069	6.32 × 10^−4^	2.77 × 10^−1^
	Benzocaine	1.69823	8.81 × 10^−4^	2.36 × 10^0^
	Beta-mangostin	1.70101	1.45 × 10^−3^	6.29 × 10^0^
	3-Hydroxycoumarin	1.70265	1.74 × 10^−3^	1.25 × 10^−1^
	Alpha-Santonin	1.70075	2.53 × 10^−3^	3.83 × 10^0^
	Guanosine 3′,5′-cyclic monophosphate	1.67239	3.40 × 10^−3^	5.20 × 10^0^
	Astragalin	1.66927	3.45 × 10^−3^	4.27 × 10^−1^
	Anabasine	1.65347	3.88 × 10^−3^	2.56 × 10^0^
	Peonidin-3-glucoside	1.65909	4.01 × 10^−3^	3.03 × 10^0^
	Methyl cinnamate	1.66713	4.44 × 10^−3^	3.73 × 10^−1^
	Agrocybenine	1.71853	4.51 × 10^−3^	3.18 × 10^1^
	Epicatechin; (+)-Epicatechin	1.64442	5.34 × 10^−3^	7.18 × 10^−1^
	Benzoic acid	1.65635	5.79 × 10^−3^	3.60 × 10^−1^
DWF0 vs. DWF6	Biochanin A	1.78505	5.99 × 10^−4^	4.95 × 10^0^
	3-(2-Hydroxyphenyl)-2-propenal	1.76743	6.69 × 10^−4^	2.56 × 10^−1^
	Benzocaine	1.75314	1.02 × 10^−3^	2.36 × 10^0^
	(+)-Abscisic acid	1.7912	1.48 × 10^−3^	2.76 × 10^0^
	Prunetin	1.74471	1.76 × 10^−3^	3.48 × 10^0^
	Sinalbin	1.77845	2.02 × 10^−3^	4.61 × 10^0^
	Diosmetin	1.7488	2.2 × 10^−3^	5.32 × 10^0^
	Alpha-Santonin	1.73205	3.51 × 10^−3^	2.76 × 10^0^
	18alpha-Glycyrrhetinic acid	1.72805	4.10 × 10^−3^	2.09 × 10^0^
	Guanosine 3′,5′-cyclic monophosphate	1.72264	5.96 × 10^−3^	4.86 × 10^0^
	Rotenone	1.71587	6.63 × 10^−3^	2.00 × 10^0^
	3-Hydroxycoumarin	1.77257	7.33 × 10^−3^	2.27 × 10^−1^
	Albiflorin	1.70167	7.47 × 10^−3^	3.61 × 10^−1^
	Myricitrin	1.75625	8.05 × 10^−3^	4.11 × 10^−1^
	Methyl cinnamate	1.72535	8.50 × 10^−3^	4.50 × 10^−1^
DWF4 vs. DWF5	Myricitrin	1.81102	5.22 × 10^−5^	3.22 × 10^−1^
	Acevaltrate	1.79949	3.63 × 10^−4^	3.62 × 10^−1^
	Methyl cinnamate	1.77422	1.58 × 10^−3^	4.20 × 10^−1^
	Allura Red AC	1.76653	1.65 × 10^−3^	4.20 × 10^−1^
	Quercitrin	1.75775	3.28 × 10^−3^	7.12 × 10^−1^
	Benzoic acid	1.76006	4.08 × 10^−3^	3.98 × 10^−1^
	Nalidixic Acid	1.75741	5.26 × 10^−3^	3.87 × 10^−1^
	Methyl rosmarinate	1.71246	5.41 × 10^−3^	6.00 × 10^−1^
	Beta-mangostin	1.76631	6.23 × 10^−3^	2.54 × 10^0^
	trans-Zeatin-riboside	1.67633	9.62 × 10^−3^	6.11 × 10^−1^
	Okanin	1.72165	1.03 × 10^−2^	5.96 × 10^−1^
	Phloretic acid; Ethylparaben; 3-(2-Hydroxyphenyl)propanoic acid	1.78558	1.15 × 10^−2^	1.05 × 10^−1^
	Dalbergioidin	1.71805	1.26 × 10^−2^	4.51 × 10^−1^
	Albiflorin	1.68098	1.29 × 10^−2^	5.10 × 10^−1^
	6(1H)-Azulenone, 2,3-dihydro-1,4-dimethyl	1.65178	1.45 × 10^−2^	4.74 × 10^−1^
DWF4 vs. DWF6	Myricitrin	1.72507	6.98 × 10^−5^	2.93 × 10^−1^
	Acevaltrate	1.71887	7.38 × 10^−5^	3.46 × 10^−1^
	Fisetin	1.70209	1.27 × 10^−3^	5.30 × 10^−1^
	Quercitrin	1.68405	1.92 × 10^−3^	6.79 × 10^−1^
	Methyl cinnamate	1.66177	2.52 × 10^−3^	5.06 × 10^−1^
	Gamabufotalin; Telocinobufagin	1.66111	3.99 × 10^−3^	4.03 × 10^−1^
	2′-Deoxyuridine; Deoxyuridine	1.64943	4.14 × 10^−3^	3.23 × 10^−1^
	Glycyrrhetic acid 3-O-mono-beta-D-glucuronide	1.64974	4.29 × 10^−3^	3.16 × 10^−1^
	Albiflorin	1.65853	5.41 × 10^−3^	3.76 × 10^−1^
	Procyanidin B2	1.6236	6.20 × 10^−3^	3.24 × 10^−1^
	(R)-Menthofuran	1.63394	6.48 × 10^−3^	6.28 × 10^−1^
	Cianidanol	1.65171	6.50 × 10^−3^	4.68 × 10^−1^
	Eurycomalactone; Ingenol	1.64399	6.73 × 10^−3^	6.13 × 10^−1^
	Allura Red AC	1.64917	7.66 × 10^−3^	2.64 × 10^−1^
	trans-Zeatin-riboside	1.61102	8.19 × 10^−3^	6.18 × 10^−1^
DWF5 vs. DWF6	Linderane	1.82323	2.06 × 10^−3^	2.28 × 10^0^
	1-Naphthylacetic acid	1.78242	3.38 × 10^−3^	3.39 × 10^−1^
	Silibinin	1.79779	5.15 × 10^−3^	2.74 × 10^−1^
	1H-Indole-2,3-dione	1.77139	5.71 × 10^−3^	5.28 × 10^−1^
	(Â±)-Jasmonic acid	1.73667	6.82 × 10^−3^	4.00 × 10^−1^
	Cleomiscosin A	1.71992	1.39 × 10^−2^	6.35 × 10^−1^
	(−)-Borneol; (+)-Borneol	1.80678	1.57 × 10^−2^	5.51× 10^−2^
	Narcissoside	1.69806	1.73 × 10^−2^	3.79 × 10^−1^
	Terpinine-4-ol	1.73342	1.87 × 10^−2^	1.66 × 10^0^
	Neogrifolin; Grifolin; Ugaxanthone	1.66903	1.99 × 10^−2^	6.04 × 10^−1^
	Cyanidin-3-O-rutinoside chloride	1.68873	2.01 × 10^−2^	3.97 × 10^−1^
	L-Gulose	1.70231	2.08 × 10^−2^	4.39 × 10^−1^
	Rhoifolin	1.73982	2.16 × 10^−2^	3.20 × 10^−1^
	Diosmetin	1.74586	2.27 × 10^−2^	4.44 × 10^−1^
	Liquiritin	1.65423	2.35 × 10^−2^	4.12 × 10^−1^

**Table 4:** The top 10 metabolic pathways enriched in hazelnut leaves.

Treatments	ID Annotation	Annotation	*p*-Value	−log(*p*-Value)
DWF0 vs. DWF4	map00941	Flavonoid biosynthesis	2.67 × 10^−2^	1.57 × 10^0^
	map00240	Pyrimidine metabolism	8.91 × 10^−2^	1.05 × 10^0^
	map00943	Isoflavonoid biosynthesis	9.94 × 10^−2^	1.00 × 10^0^
	map00626	Naphthalene degradation	1.06 × 10^−1^	9.74 × 10^−1^
	map04744	Phototransduction	1.06 × 10^−1^	9.74 × 10^−1^
	map04216	Ferroptosis	1.06 × 10^−1^	9.74 × 10^−1^
	map00380	Tryptophan metabolism	1.23 × 10^−1^	9.10 × 10^−1^
	map04977	Vitamin digestion and absorption	1.25 × 10^−1^	9.03 × 10^−1^
	map01100	Metabolic pathways	1.28 × 10^−1^	8.93 × 10^−1^
	map00944	Flavone and flavonol biosynthesis	1.49 × 10^−1^	8.28 × 10^−1^
DWF0 vs. DWF5	map00232	Caffeine metabolism	8.35 × 10^−2^	1.08 × 10^0^
	map07216	Catecholamine transferase inhibitors	1.02 × 10^−1^	9.90 × 10^−1^
	map04966	Collecting duct acid secretion	1.02 × 10^−1^	9.90 × 10^−1^
	map05120	Epithelial cell signaling in Helicobacter pylori infection	1.02 × 10^−1^	9.90 × 10^−1^
	map07034	Eicosanoids	1.02 × 10^−1^	9.90 × 10^−1^
	map00626	Naphthalene degradation	1.02 × 10^−1^	9.90 × 10^−1^
	map00910	Nitrogen metabolism	1.02 × 10^−1^	9.90 × 10^−1^
	map04744	Phototransduction	1.02 × 10^−1^	9.90 × 10^−1^
	map00230	Purine metabolism	1.13 × 10^−1^	9.47 × 10^−1^
	map00944	Flavone and flavonol biosynthesis	1.37 × 10^−1^	8.64 × 10^−1^
DWF0 vs. DWF6	map00943	Isoflavonoid biosynthesis	2.37 × 10^−4^	3.63 × 10^0^
	map01100	Metabolic pathways	6.17 × 10^−2^	1.21 × 10^0^
	map07034	Eicosanoids	6.46 × 10^−2^	1.19 × 10^0^
	map00626	Naphthalene degradation	6.46 × 10^−2^	1.19 × 10^0^
	map04744	Phototransduction	6.46 × 10^−2^	1.19 × 10^0^
	map01061	Biosynthesis of phenylpropanoids	8.98 × 10^−2^	1.05 × 10^0^
	map00400	Phenylalanine, tyrosine, and tryptophan biosynthesis	1.09 × 10^−1^	9.64 × 10^−1^
	map04713	Circadian entrainment	1.25 × 10^−1^	9.02 × 10^−1^
	map04976	Bile secretion	1.25 × 10^−1^	9.02 × 10^−1^
	map04921	Oxytocin signaling pathway	1.25 × 10^−1^	9.02 × 10^−1^
DWF4 vs. DWF5	map00310	Lysine degradation	2.87 × 10^−2^	1.54 × 10^0^
	map07216	Catecholamine transferase inhibitors	4.79 × 10^−2^	1.32 × 10^0^
	map00270	Cysteine and methionine metabolism	9.37 × 10^−2^	1.03 × 10^0^
	map00908	Zeatin biosynthesis	1.37 × 10^−1^	8.62 × 10^−1^
	map00941	Flavonoid biosynthesis	1.64 × 10^−1^	7.86 × 10^−1^
	map00622	Xylene degradation	1.79 × 10^−1^	7.46 × 10^−1^
	map00240	Pyrimidine metabolism	2.19 × 10^−1^	6.59 × 10^−1^
	map00945	Stilbenoid, diarylheptanoid, and gingerol biosynthesis	2.19 × 10^−1^	6.59 × 10^−1^
	map00232	Caffeine metabolism	2.19 × 10^−1^	6.59 × 10^−1^
	map01060	Biosynthesis of plant secondary metabolites	2.28 × 10^−1^	6.42 × 10^−1^
DWF4 vs. DWF6	map00760	Nicotinate and nicotinamide metabolism	5.64 × 10^−2^	1.25 × 10^0^
	map00562	Inositol phosphate metabolism	5.82 × 10^−2^	1.23 × 10^0^
	map04070	Phosphatidylinositol signaling system	5.82 × 10^−2^	1.23 × 10^0^
	map00941	Flavonoid biosynthesis	8.33 × 10^−2^	1.08 × 10^0^
	map00521	Streptomycin biosynthesis	1.13 × 10^−1^	9.46 × 10^−1^
	map01062	Biosynthesis of terpenoids and steroids	1.29 × 10^−1^	8.90 × 10^−1^
	map00052	Galactose metabolism	1.65 × 10^−1^	7.82 × 10^−1^
	map01250	Biosynthesis of nucleotide sugars	1.65 × 10^−1^	7.82 × 10^−1^
	map00053	Ascorbate and aldarate metabolism	1.65 × 10^−1^	7.82 × 10^−1^
	map00830	Retinol metabolism	1.65 × 10^−1^	7.82 × 10^−1^
DWF5 vs. DWF6	map00944	Flavone and flavonol biosynthesis	2.47 × 10^−2^	1.61 × 10^0^
	map00592	alpha-Linolenic acid metabolism	6.04 × 10^−2^	1.22 × 10^0^
	map04075	Plant hormone signal transduction	6.04 × 10^−2^	1.22 × 10^0^
	map00053	Ascorbate and aldarate metabolism	6.04 × 10^−2^	1.22 × 10^0^
	map00760	Nicotinate and nicotinamide metabolism	1.36 × 10^−1^	8.66 × 10^−1^
	map01060	Biosynthesis of plant secondary metabolites	1.53 × 10^−1^	8.15 × 10^−1^
	map01070	Biosynthesis of plant hormones	2.40 × 10^−1^	6.19 × 10^−1^
	map00941	Flavonoid biosynthesis	4.80 × 10^−1^	3.19 × 10^−1^
	map01110	Biosynthesis of secondary metabolites	5.74 × 10^−1^	2.41 × 10^−1^
	map01061	Biosynthesis of phenylpropanoids	5.79 × 10^−1^	2.37 × 10^−1^

**Table 5:** Metabolites corresponding to flavonoid metabolic pathways in hazelnut leaves.

Treatments	Metabolic Pathway		
	Flavonoid Biosynthesis	Isoflavonoid Biosynthesis	Flavone and Flavonol Biosynthesis
DWF0 vs. DWF4	Tricetin Pinocembrin Eriodictyol Garbanzol Phlorizin Luteolin Apigenin	Calycosin Biochanin A Apigenin	Quercitrin Luteolin Apigenin
DWF0 vs. DWF5	Eriodictyol Garbanzol Apigenin	Prunetin Apigenin	Astragalin Rhoifolin Apigenin
DWF0 vs. DWF6	Eriodictyol Apigenin	Calycosin Rotenone Prunetin Biochanin A Apigenin	Apigenin
DWF4 vs. DWF5	Tricetin Pinocembrin Cianidanol	Prunetin	Quercitrin
DWF4 vs. DWF6	Tricetin Pinocembrin Cianidanol Phlorizin		Quercitrin
DWF5 vs. DWF6	Kaempferol		Rhoifolin Kaempferol

## Data Availability

Data will be made available on request.
